# Editorial: Advancements and challenges in CAR-T cell therapy for cancer treatment

**DOI:** 10.3389/fphar.2026.1820259

**Published:** 2026-04-09

**Authors:** Alexander T. Sougiannis, Angelica Meneses Acosta, Leonardo M. R. Ferreira

**Affiliations:** 1 Wexner Medical Center, The Ohio State University, Columbus, OH, United States; 2 Universidad Autonoma del Estado de Morelos, Cuernavaca, Mexico; 3 Department of Pharmacology and Immunology, Medical University of South Carolina, Charleston, SC, United States; 4 Hollings Cancer Center, Medical University of South Carolina, Charleston, SC, United States

**Keywords:** CAR (chimeric antigen receptor) T cells, cellular engineering, immunoengineering, personalized medicine, T cell, translational medicine, cancer, solid tumor

CAR-T cell therapy represents an innovative form of immunotherapy, wherein T cells are genetically modified to recognize and target specific cancer cells, offering a personalized and targeted approach to cancer treatment. A chimeric antigen receptor (CAR) is a specially designed molecule comprised of an extracellular antigen-binding domain and an intracellular signaling domain, which together drive CAR-T cell activation upon antigen recognition. This breakthrough therapy has shown impressive clinical outcomes, particularly in hematological malignancies, with a significant number of patients achieving complete remission after failing conventional therapies. However, extending the success of CAR-T cell therapy to solid tumors presents unique challenges and opportunities. This Research Topic brings together articles addressing the distinct challenges and advancements in CAR-T cell therapy for blood cancers and solid tumors, providing comprehensive insights into the current state and future directions of this promising cancer treatment modality.

Collectively, the seven articles in this Research Topic highlight both the remarkable progress achieved in adoptive cell therapies and the complex biological, clinical, and translational barriers that must be overcome to broaden their impact. A recurring theme across these contributions is that while CAR-T cell therapy has matured into a validated therapeutic platform, its future success depends on deeper mechanistic understanding, improved patient stratification, thoughtful combination strategies, and innovations in cellular engineering.

One of the foundational yet often underappreciated challenges in CAR-T development is immunogenicity. Alfar et al. provide a timely and comprehensive review of clinical evidence demonstrating that CAR-T cells, like other biologic therapies, can elicit immune responses that compromise safety and efficacy. Anti-CAR antibodies and cellular immune responses can alter CAR-T pharmacokinetics, limit persistence, and contribute to treatment failure. By systematically analyzing immunogenicity data from FDA- and EMA-approved CAR-T products, this review underscores the need for standardized assays, robust preclinical models, and proactive monitoring strategies during clinical development. As CAR constructs become increasingly complex—incorporating non-human domains, gene edits, and synthetic circuits—addressing immunogenicity early will be critical to ensuring durable clinical benefit.

Despite impressive initial responses, relapse after CAR-T therapy remains a significant clinical problem, particularly in multiple myeloma. Min et al. focus on the post–CAR-T landscape in relapsed or refractory multiple myeloma, where antigen loss, T cell exhaustion, and tumor microenvironmental pressures drive disease recurrence. Their review highlights emerging salvage strategies, including repeat CAR-T infusion, bispecific antibodies, antibody–drug conjugates, and combination regimens. Importantly, this work emphasizes that CAR-T therapy should not be viewed as a terminal intervention, but rather as one component of a dynamic treatment continuum. Therefore, understanding mechanisms of resistance and tailoring post–CAR-T interventions accordingly will be essential to extending therapeutic benefit.

While hematologic malignancies continue to benefit most from CAR-T therapy, expanding these successes to solid tumors remains a central challenge for the field. Zhao et al. offer a broad and insightful overview of cell-based immunotherapies for solid tumors, including CAR-T, CAR-NK, and TCR-engineered T cells. Their analysis highlights shared obstacles such as antigen heterogeneity, physical barriers to infiltration, and profoundly immunosuppressive tumor microenvironments. At the same time, the authors emphasize emerging opportunities, including multi-antigen targeting, gene editing, synthetic biology, and off-the-shelf products. This review positions CAR-T therapy within a broader ecosystem of cellular therapies and reinforces the idea that no single modality will be universally effective; rather, rational design and personalization will be key.

Complementing this systems-level perspective, Liu et al. focus on adoptive cell therapy more broadly, with particular emphasis on combination strategies and biomarkers. Their review highlights how combining ACT with chemotherapy, radiotherapy, immune checkpoint blockade, or targeted agents can enhance efficacy while mitigating toxicity. Equally important is their discussion of biomarkers, which are increasingly critical for patient selection, product characterization, toxicity prediction, and response monitoring. As CAR-T therapies move into earlier lines of treatment and more diverse patient populations, biomarker-driven decision-making will be essential for maximizing benefit while minimizing risk.

Several articles in this Research Topic address specific strategies to overcome barriers unique to solid tumors. Kumar et al. provide compelling preclinical evidence that engineering CAR-T cells with chemokine receptors can significantly improve tumor trafficking and efficacy in ovarian cancer. By identifying CCL2 as a commonly expressed chemokine in ovarian tumors and arming CAR-T cells with its cognate receptor CCR2, the authors demonstrate enhanced peritoneal homing and prolonged survival *in vivo*. This work exemplifies how rational engineering informed by tumor biology can directly address one of the most persistent limitations of CAR-T therapy in solid malignancies: insufficient infiltration.

As CAR-T therapies become more widely used and integrated into multimodal treatment regimens, careful attention to safety and rare toxicities is increasingly important. The case report by Li et al. describing Purtscher-like retinopathy following CAR-T therapy and subsequent allogeneic hematopoietic stem cell transplantation highlights the cumulative endothelial injury that can arise from intensive immunotherapy. This report underscores the need for vigilant post-treatment monitoring, particularly in patients receiving sequential or combination immune-based therapies. It also illustrates how immune activation, infection, and vascular injury can intersect in unexpected ways, reinforcing the importance of interdisciplinary clinical awareness.

Finally, Zhou et al. focus on advancing CAR-T therapy in prostate cancer, a disease where immunotherapy has historically shown limited success. Their review synthesizes current knowledge of prostate cancer–specific antigens, tumor microenvironmental barriers, and emerging CAR engineering strategies. Approaches such as armored CAR-T cells, metabolic reprogramming, checkpoint disruption, and multi-antigen targeting are highlighted as promising avenues to enhance efficacy. Although durable remissions in prostate cancer remain rare, the progress summarized in this article underscores that rational design and combination strategies may yet unlock the potential of CAR-T therapy in this challenging disease.

Taken together, the articles in this Research Topic paint only a small picture of the potential of CAR-T cell therapy at a pivotal moment in its evolution. The field has moved beyond proof-of-concept and now faces the more complex task of optimization—improving durability, expanding indications, managing toxicity, and ensuring equitable access. Advances in basic immunology, bioengineering, and clinical trial design are converging to address these challenges. As illustrated by the contributions in this Research Topic, the future of CAR-T therapy will be shaped not by a single breakthrough, but by the integration of mechanistic insight, technological innovation, and clinical experience. Through continued interdisciplinary collaboration, CAR-T cell therapy has the potential to transform outcomes not only for patients with blood cancers, but also for those with historically intractable solid tumors.

